# A knowledge-based Bayesian network reveals shared prediction errors with physicians in Gamma Knife radiosurgery

**DOI:** 10.3389/fnins.2026.1864936

**Published:** 2026-08-17

**Authors:** Yusuke Uchiyama, Soichiro Fujiki, Kensaku Nomoto, Toshihiro Kawase, Kyoko Aoyagi, Yoshinori Higuchi, Jun Nakano, Kenji Kansaku

**Affiliations:** 1 Department of Physiology, Dokkyo Medical University, Graduate School of Medicine, Mibu, Tochigi, Japan; 2 Department of Information and Communication Engineering, Tokyo Denki University, Adachi-ku, Tokyo, Japan; 3 Department of Neurosurgery, Chiba Cerebral and Cardiovascular Center, Ichihara, Chiba, Japan; 4 Department of Neurosurgery, Chiba University Graduate School of Medicine, Chiba, Japan; 5 Department of Information and Computer Science, Kanazawa Institute of Technology, Nonoichi, Ishikawa, Japan

**Keywords:** Bayesian network, clinical reasoning, decision making, Gamma Knife radiosurgery, prediction error

## Abstract

Although Artificial Intelligence (AI) has shown strong predictive performance in medicine, its role in providing cognitive support remains unclear. We developed a knowledge-based expert system using a Bayesian Network (BN) to explicitly model an expert neurosurgeon's clinical reasoning for predicting outcomes of Gamma Knife radiosurgery. The BN structure was constructed from elicited expert knowledge and encoded as a directed acyclic (DAG) graph that represents clinically relevant variables and their interrelationships. Model parameters were estimated using a clinical dataset to predict patient overall survival (OS). In parallel, physicians independently estimated outcomes for the same patients, enabling a direct comparison of predictive performance and error characteristics. The BN captured clinically plausible reasoning patterns and produced less skewed prediction errors than physicians, suggesting partial mitigation of individual cognitive bias. However, both the BN and physicians exhibited a heavy-tailed error distribution, systematically failing to predict patients with unexpectedly long-term survival. This convergence of error patterns indicates that both human and model-based predictions are constrained by unobserved or unmodeled factors, limiting their ability to capture exceptional outcomes. These findings highlight a limitation of predictive paradigms based on current expert knowledge and suggest the need for iterative refinement of knowledge-based models. Incorporating additional expert knowledge and updating the underlying structure may help identify previously unmodeled relationships and reduce unexplained variance. Taken together, our results position knowledge-based BNs not only as interpretable predictive tools, but also as evolving frameworks for integrating clinical knowledge and probing the shared limits of human-AI decision-making, highlighting their potential as computational cognitive neuroprosthetic systems that aim to augment physicians' clinical reasoning under uncertainty.

## 1 Introduction

The advent of advanced Artificial Intelligence (AI) has ushered in a new era of predictive modeling, with significant applications in medicine (Esteva et al., 2019; Shen et al., 2017; Litjens et al., 2017; Greenspan et al., 2016; Fu et al., 2020). For instance, AI technologies assist in detecting disease predictors from medical charts (Rajkomar et al., 2019, 2018; Liang et al., 2020), interpreting radiographic images (Monshi et al., 2020; Suk et al., 2016; Sarraf and Tofighi, 2016), and assessing rehabilitation progress (Kawahara et al., 2021; Khodatars et al., 2021; Secinaro et al., 2021; Kumar et al., 2023). However, the adoption of these powerful tools in clinical practice is often approached with caution. A primary obstacle is their “black box” nature. Clinicians are ethically and professionally obligated to provide clear justifications for their diagnostic and treatment decisions. Unlike traditional rule-based expert systems that rely on explicit logic, many modern AI models, despite their high accuracy, cannot explain the reasons for their outputs. This lack of transparency and interpretability is a critical barrier to their adoption as trusted decision-support tools in clinical settings (Reddy, 2022; Zhang et al., 2022; Amann et al., 2020). In this context, clinical decision-support systems can be viewed as a form of cognitive neuroprosthesis designed to augment human clinical reasoning under uncertainty. Moreover, even when predictive performance is high, it remains unclear whether such models truly complement or merely replicate the limitations of human clinical decision-making.

Bayesian Networks (BNs) provide a powerful framework for developing such knowledge-driven expert systems (Heckerman, 2008; Jensen and Nielsen, 2007; Friedman and Koller, 2003; Bielza and Larranaga, 2014; Fenton and Neil, 2018; Shen et al., 2022). As a class of probabilistic graphical models, BNs represent a set of variables and their probabilistic relationships, more precisely their conditional independencies, using a directed acyclic graph (DAG). This graphical structure provides an intuitive and explicit map of the interrelationships among factors, mirroring the mental models of human experts (Waldmann and Martignon, 2022; Marcot and Penman, 2019; Velikova et al., 2014). Crucially, the network structure of a BN can be constructed directly from experts' domain knowledge, allowing for the formal encoding of their experience and understanding of clinical relationships. The model's parameters can then be refined using clinical data, creating a synergistic system that integrates expert knowledge with data-driven evidence. This approach produces a model that is not only predictive but also inherently interpretable because its reasoning process is transparent and aligned with established expertise. The property makes BNs particularly suitable for examining not only predictive performance, but also the nature of agreement and divergence between human and model-based reasoning.

This study presents the design and application of a BN-based expert system for predicting patient overall survival (OS). Recognizing that expert clinical judgment is a complex synthesis of knowledge and experience, we sought to develop a system that emulates this process. The core of our methodology involved collaborating with an expert neurosurgeon to construct a BN structure that explicitly models the interrelationships among key clinical and treatment variables. This knowledge-based graph was then applied to a clinical dataset to learn probabilistic relationships and predict patient survival (Flores et al., 2011). We then evaluated our system's predictive performance and, critically, compared its decision-making characteristics with the predictions of six other physicians. Rather than focusing solely on accuracy, we aimed to analyze the structure of prediction errors that are shared between the model and physicians. Our focus is on demonstrating that a purely knowledge-driven model structure, informed by a single expert, can effectively simulate the nuanced reasoning of a human specialist. This approach contrasts with many hybrid models by intentionally isolating the contribution of expert knowledge, thereby creating a more direct simulation of a physician's cognitive process. Through this comparison, we further explore whether a knowledge-based model can mitigate or instead reproduce the systematic limitations in human prediction. The choice of a BN is further motivated by its conceptual alignment with contemporary neuroscience, particularly the “Bayesian brain” hypothesis. This influential theory holds that the brain represents knowledge probabilistically and performs computations equivalent to Bayesian inference to make optimal decisions under uncertainty (Knill and Pouget, 2004; Doya, 2007). While initially focused on sensory-motor functions, this framework has been extended to explain higher-level cognitive processes such as clinical reasoning and knowledge acquisition, which are central to expert judgment (Tenenbaum et al., 2011). Modern theories, like the free-energy principle, cast the brain as a sophisticated prediction machine that constantly updates its internal probabilistic models of the world to minimize surprise or prediction error (Friston, 2010). In this context, a BN serves as a compelling computational analog to the hypothesized mechanisms underlying expert cognition. By building a BN from a physician's explicit knowledge, our study aims not only to create a predictive tool but also a functional model that reflects the very structure of expert cognitive reasoning as understood by modern neuroscience. Importantly, this perspective also allows interpreting model-human discrepancies as signals to update internal representations, suggesting a pathway toward the iterative refinement of clinical knowledge structures.

## 2 Methods

### 2.1 Data description

In this study, we reanalyzed data from our observational cohort study (Higuchi et al., 2017, 2018). This retrospective study was approved by the institutional review board of the Chiba Cerebral and Cardiovascular Center Institutional Review Board (IRB) (IRB number: #456). All methods were performed in accordance with the institutional guidelines. The dataset includes data from participants with several brain metastases from the Chiba Cerebral and Cardiovascular Center in Japan. Patients were eligible for inclusion if, at the time of stereotactic radiosurgery, they had newly diagnosed brain metastases confirmed by contrast-enhanced magnetic resonance imaging (MRI) within 6 weeks before the procedure, had no findings of leptomeningeal dissemination, and had a Karnofsky performance status (KPS) score of 70 or higher, or, in patients with a KPS score of less than 70, a reasonable expectation of improvement in neurological function with stereotactic radiosurgery. Patients with all types of primary malignant tumors–except sarcoma and lymphoma, which are not solid cancers–were eligible. Patients were excluded if they had two or more primary malignant tumors, were pregnant or breastfeeding, had a prior diagnosis of any psychological disorder, had contraindications to MRI or gadolinium use, or had prior surgery or irradiation of the skull or brain. Patients with confirmed death events were included in the analysis (*N* = 1, 738). No missing values were present in any of the variables used for modeling (confirmed by data audit). The dataset was split into a training set (*N* = 1, 390, 80%) and a test set (*N* = 350, 20%) using a single random split with no stratification.

**Figure 1:**
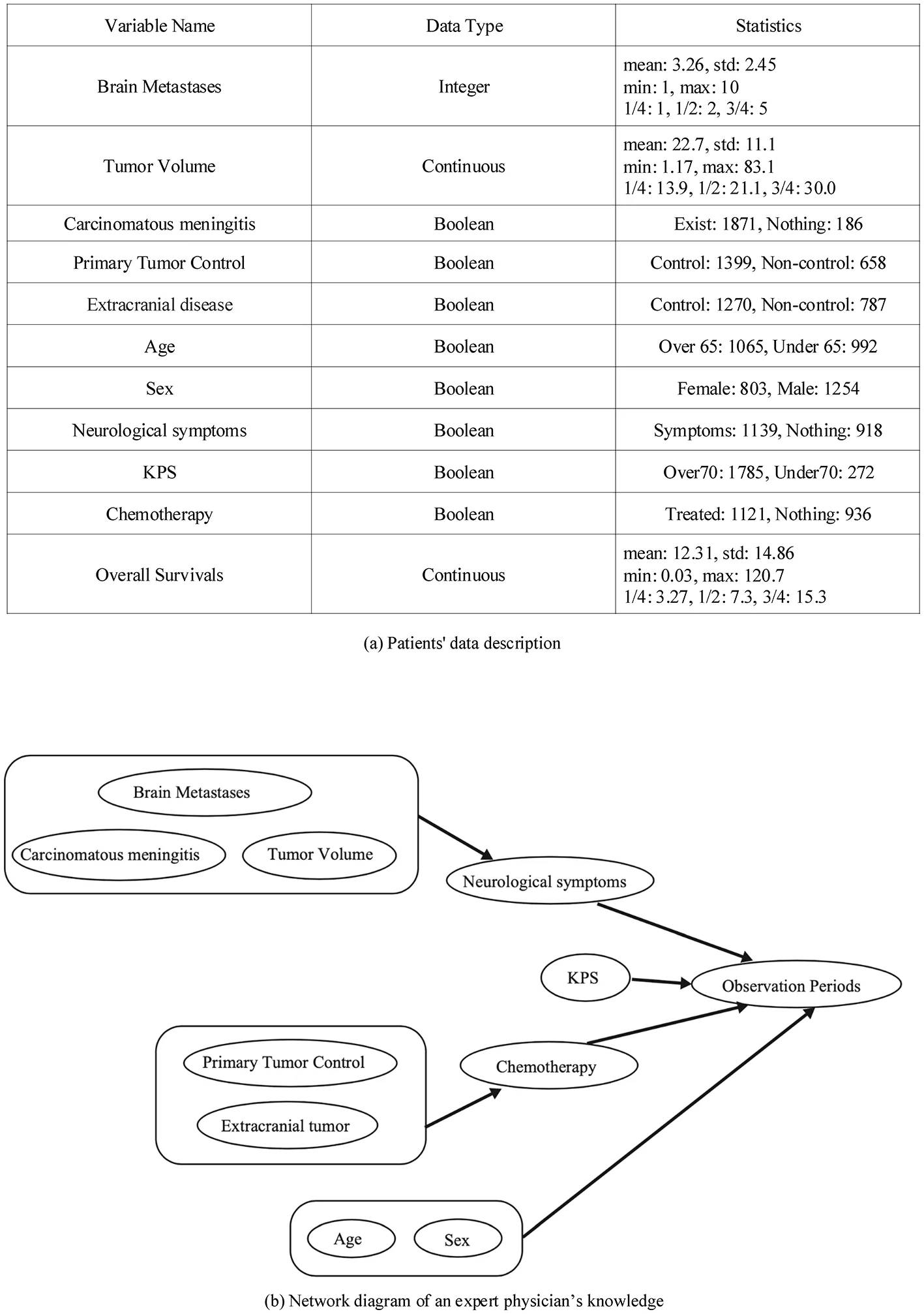
**(a)** Statistical description of the patients' clinical data. **(b)** Basis graph network of the BN prescribed by the expert physician.

The observed dataset is summarized in Figure 1a. The brain metastases were given as integer data, the tumor volume and OS (months) were observed as continuous data, and others were measured as Boolean data.

### 2.2 Bayesian network

BNs represent systems in terms of random variables and model the conditional independencies of factors considered. BNs are graphically represented by DAGs, whose respective nodes and arcs represent random variables and direct dependencies. In this research, the initial DAG structure was identified using a hill-climbing algorithm and subsequently adjusted by an expert physician to align with clinical reality based on previous studies (Sperduto et al., 2010, 2012; Higuchi et al., 2017). Subsequently, the other four physicians confirmed and validated the DAG based on their clinical knowledge.

Suppose **X** = {*X*_1_, *X*_2_, ⋯ , *X*_*n*_} is the set of random variables on nodes. The corresponding joint probability *P*(**X**) is given as the product of conditional probabilities of each node:


$$\begin{array}{l} P\left(\text{X}\right)=\overset{n}{\underset{i=1}{\prod }}P\left(X_{i}|\text{Pa}\left(X_{i}\right)\right) \end{array}$$ (1)

where Pa(*X*_*i*_) is the set of random variables on parent nodes for *X*_*i*_. The structures of DAGs are determined by expert knowledge and the given dataset simultaneously. The former have been developed using numerical algorithms that involve Bayes' rule. On the other hand, we construct DAGs to incorporate interrelations between nodes with expert knowledge.

In learning tasks, the conditional probabilities of BNs, as parameters, are estimated from a given dataset. Let *D* = {*D*_1_, *D*_2_, ⋯ , *D*_*m*_} be the given dataset; a likelihood function of a BN is expressed as


$$\begin{array}{l} L\left(\theta \right)=P\left(D|\theta \right)\\ \;=\overset{m}{\underset{j=1}{\prod }}P\left(D_{j}|\theta \right), \end{array}$$ (2)

where θ denotes the parameters of the BN. For *L*(θ) in Equation 2, the maximum likelihood estimator with a regularization term provides the candidate parameter as follows:


$$\begin{array}{l} \theta^{*}=\arg max_{\theta }L\left(\theta \right)+\lambda g\left(\theta \right), \end{array}$$ (3)

where *g*(θ) is a regularization term and λ is a Lagrangian multiplier. In the literature of Bayesian modeling, parameters of BNs are also regarded as random variables. Given the prior of the parameters by *P*(θ), the corresponding posterior is derived from Bayes' theorem as


$$\begin{array}{l} P\left(\theta |D\right)=\frac{P\left(D|\theta \right)P\left(\theta \right)}{P\left(D\right)}. \end{array}$$ (4)

The posterior *P*(θ∣*D*) in Equation 4 is often approximately estimated by variational inference or Markov chain Monte Carlo methods. Maximum a posteriori estimator


$$\begin{array}{l} \theta^{*}=\arg max_{\theta }P\left(D|\theta \right)P\left(\theta \right) \end{array}$$ (5)

is also used as a candidate for the parameters. In this study, model parameters were estimated using the Bayesian Dirichlet equivalent uniform (BDeu) estimator with equivalent sample size = 10, implemented via the DiscreteBayesianNetwork class in pgmpy (v1.0.0). This corresponds to a Dirichlet prior with uniform pseudo-counts and serves as the regularization term λ*g*(θ) in Equation 3. Tumor diameter and OS were each discretized into 10 equal-frequency (quantile) bins. Bin boundaries were determined from the full dataset and applied consistently across all evaluations. The 10-bin choice balances granularity with sample size per bin.

With estimated BN parameters in Equations 3 and 5, a conditional probability distribution *P*(*Y*∣*X*) for a factor X and an outcome Y is calculated by marginalization for Equation 1. Based on the conditional probability distribution, we define a statistical relevance factor which evaluates the magnitude of sensitivity of an input *X* to output *Y* as


$$\begin{array}{l} CF\left(X\to Y\right)=\underset{x^{\prime }}{\sum }|\frac{1}{N_{X}-1}\underset{x\neq x^{\prime }}{\sum }\underset{Y}{\sum }YP\left(Y|X=x\right)\\ \;-\underset{Y}{\sum }YP\left(Y|X=x^{\prime }\right)|. \end{array}$$ (6)

For binary variable *X* = 0, 1, *CF*(*X*→*Y*) in Equation 6 is reduced as


$$\begin{array}{l} CF\left(X\to Y\right)=|\underset{Y}{\sum }YP\left(Y|X=0\right)-\underset{Y}{\sum }YP\left(Y|X=1\right)|. \end{array}$$ (7)

For continuous output *Y*, summations are replaced with integrals when calculating the statistical relevance factor. A statistical relevance score in Equation 7 *CF*(*X*→*Y*)≥1.0 was adopted as the threshold for clinical significance, corresponding to an expected absolute shift of at least 1 month in predicted OS when variable X changes between its states. This threshold was agreed upon with the expert neurosurgeon as the minimum clinically meaningful difference for treatment planning.

### 2.3 Generalized hyperbolic distribution

The generalized hyperbolic (GH) distribution (Barndorff-Nielsen and Halgreen, 1977) is a unimodal and asymmetric probability density function defined by


$$\begin{array}{l} p\left(x;\mu ,\sigma ,\lambda ,\alpha ,\beta \right)=\frac{\sigma^{\lambda }K_{\lambda -1}\left(\sqrt{\alpha^{2}-\beta^{2}}\right)}{2^{\left(1+\lambda \right)/2}\pi^{1/2}K_{\lambda }\left(\sqrt{\alpha^{2}}\right)}\exp\left(\beta \left(x-\mu \right)\right)\\ \;\times {\left(\frac{\alpha }{\sqrt{\alpha^{2}-\beta^{2}}}\right)}^{\lambda }I_{\lambda }\left(\sigma \sqrt{\alpha^{2}-{\left(x-\mu \right)}^{2}}\right), \end{array}$$ (8)

where, *K*_ν_(·) is the modified Bessel function of the second kind of order ν, *I*_ν_(·) is the modified Bessel function of the first kind of order ν, α is a real-valued parameter that determines the skewness of the distribution, and β is a real-valued parameter that affects the kurtosis of the distribution. The location parameter μ represents the horizontal shift of the distribution, the scale parameter σ controls the spread or scale of the distribution, and the shape parameter λ governs the shape of the tails and determines the distribution's heaviness or lightness. The GH family in Equation 8 was selected because it is one of the most flexible parametric classes of distributions and subsumes the normal, Student-*t*, hyperbolic, and normal-inverse Gaussian distributions as special cases, allowing unbiased characterization of both skewness and heavy-tail behavior without imposing structural assumptions on the error distribution.

The underlying error distribution was unknown a priori; assuming a simpler distribution (e.g., Gaussian or Student t) without empirical justification would risk model bias. GH parameters were estimated by maximum likelihood using the scipy.stats.genhyperbolic (v1.16.3) implementation with a custom Nelder-Mead optimizer. Goodness-of-fit was assessed using a one-sample Kolmogorov-Smirnov (KS) test applied to each physician's residuals. Because six simultaneous tests were conducted, unadjusted p-values were reported, and the Bonferroni-adjusted significance threshold (α_adj_ = 0.05/6 = 0.0083) was also considered.

### 2.4 Physician's predictions

To compare with the prediction error distribution, we provided six physicians with the test dataset, which is also used to evaluate the performance of the BN, to predict patients' OS. Prognostic assessments were performed independently by board-certified neurosurgeons. The participating neurosurgeons had 6-25 years of clinical experience. None of the evaluators had been involved in the treatment or management of the study patients. All physicians were blinded to the actual clinical outcomes and assessed each case independently using the same standardized clinical information under identical conditions. We then collected the physicians' prediction results and fit the prediction error distribution using the GH distributions. Furthermore, we evaluated root mean square error (RMSE), mean absolute error (MAE), mean absolute percentage error (MAPE), and coefficient of determination (R^2^) for the physicians and compared them with those of the BN.

### 2.5 Comparative analysis with machine learning baselines

To evaluate the predictive performance of the proposed model, we implemented several standard machine learning baselines for comparison, including decision tree, random forest, support vector machine, and multi-layer perceptron. The predictive accuracy of each model was assessed using RMSE and MAE. All ML models were implemented using scikit-learn (v.1.7.2). For each model, hyperparameter tuning was performed, and the final parameters were selected once the evaluation metrics converged to ensure optimal performance for the comparison as shown in Table 1. To assess model stability and obtain confidence intervals for performance metrics, a repeated 5-fold cross-validation (CV) experiment was conducted (n_repeats = 10, 50 folds total. RMSE, MAE, MAPE, and R^2^ were computed at each fold; 95% confidence intervals were derived using a Student's *t*-distribution (df = 49).

**Table 1:** ML hyperparameter configurations.

Model	Hyperparameters	Selection criterion
Decision Tree	Maximum tree depth: 2	Convergence of RMSE
Random Forest	Maximum tree depth: 3, Number of trees: 100	Convergence of RMSE
SVM	Kernel: radial basis function, Regularization strength (*C*): 1.0, Insensitive error margin (ε): 0.1 (sklearn defaults)	Default; convergence confirmed
MLP	Hidden layer: 1 hidden layer with 200 units, Maximum training iterations: 500, Convergence tolerance: 0.01	Convergence of training loss

## 3 Results

We constructed a BN based on the domain knowledge of an expert physician; we asked him to construct a graph network about relationships between factors in the clinical dataset, which was used for the BN of other physicians in this research. Then, four physicians confirmed and validated the graph network based on their clinical knowledge. Given a basis graph network by the expert physician as shown in Figure 1b, we trained the parameters of the BN using maximum likelihood with regularized terms in the form of a binary distribution for 80% of the patients' dataset. To train the BN model, we set all the variables as discrete, where tumor volume and OS were divided into two and five bins, respectively. Then the BN is trained as a discrete variable prediction model, where the outcome is the OS.

From a clinical perspective, prediction models should provide a plausible explanation for diagnosis and analyze and compare physicians' reasoning patterns, rather than prioritizing prediction accuracy, because of patient accountability. As an indicator of diagnostic tendency, we evaluated the shape of the prediction error distribution using a parametric probability density function for the remaining dataset. To estimate the order of skewness and kurtosis using parameters, we used the generalized hyperbolic distribution, which expresses a wide range of probability distributions through its parameter values, as a statistical model of the prediction error distribution. Then we used the Wilcoxon signed-rank test for the fitted parameter values of the prediction error distribution to validate the prediction tendency of physicians.

**Figure 2:**
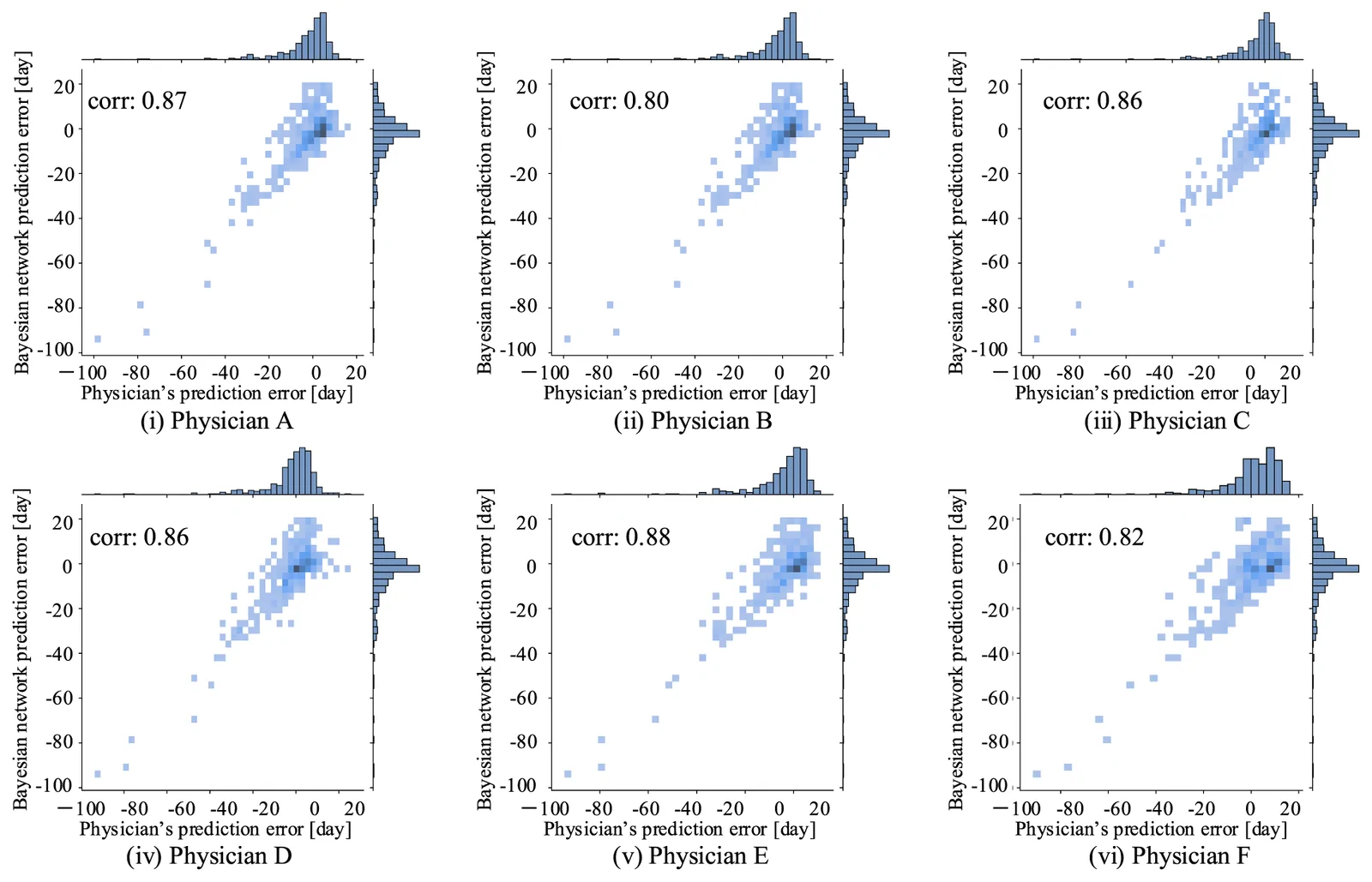
Correlation scatter plots of prediction error distributions of the BN and the physicians.

Figure 2 shows scatter plots of prediction error distributions of the BN vs. each of the six physicians. The prediction errors of both physicians and the BN were fitted by GH distributions. In all physicians' distributions, skewed and heavy-tailed shapes were observed, indicating that physicians tended to underestimate survival time (positive location parameter). Fitted GH distribution parameters for all physicians are reported in Table 2. We used the Wilcoxon signed-rank test (Wilcoxon, 1992) for the sign of each parameter of six physicians' prediction error distributions. As a result, *p*-values indicated *p* = 0.031 for positive location values (μ) and negative asymmetric values (β), confirming a systematic tendency of physicians to underestimate OS (i.e., predict shorter survival than observed). The asymmetry parameters were consistently negative across all physicians but did not reach significance after accounting for degenerate cases (*p* = 0.063). The location value of the GH distribution is regarded as an offset of outcome prediction, namely the cognitive bias of the physicians. In other words, the physicians predicted the patients' survival times to be shorter than actual values. Contrary to the skewed distributions of the physicians' prediction error, the BN's prediction error distribution was less skewed, and the location value and asymmetric value of the estimated generalized hyperbolic distribution were -0.305 and -0.086, suggesting partial mitigation of individual cognitive bias. Goodness-of-fit of the GH distributions was assessed by the KS test; results are reported in Table 3. Because six physician-specific tests were conducted, the Bonferroni-adjusted significance threshold (α_adj_ = 0.0083) was also considered.

**Table 2:** Fitted GH distribution parameters for physicians.

Doctor	μ	β	λ	α	σ
Physician A	5.220	−0.908	−0.469	1.076	5.092
Physician B	23.281	−1.30 × 10^−5^	3.445	1.33 × 10^−5^	1.67 × 10^−6^
Physician C	14.664	−22.856	−5.257	22.856	7.053
Physician D	3.880	−0.449	−0.483	0.608	5.502
Physician E	4.574	−1.359	−0.543	1.514	4.779
Physician F	22.390	−202.478	−4.757	202.478	0.828

**Table 3:** KS goodness-of-fit test results for the fitted GH distributions.

Doctor	KS statistic	*p*-value	Sig. (α=0.05)	Sig. (Bonferroni)	Note
Physician A	0.0190	0.856	No	No	—
Physician B	N/A	N/A	N/A	N/A	Degenerate (σ = 1.67 × 10^−6^≈0)
Physician C	N/A	N/A	N/A	N/A	Degenerate (α≈|β| = 22.856)
Physician D	0.0296	0.338	No	No	—
Physician E	0.0304	0.306	No	No	—
Physician F	0.0277	0.420	No	No	—

With the BN, we performed statistical relevance analysis of the outcome prediction for patients undergoing Gamma Knife radiosurgery. Statistical relevance scores of the factors of the BN are shown in descending order in Table 4. Factors such as Extracranial tumor, Primary Tumor Control, and KPS yielded scores greater than 1.0, consistent with the expert neurosurgeon's clinical knowledge. A threshold of 1.0 was adopted as the minimum clinically significant level, corresponding to an expected shift of ≥1 month in predicted OS when the factor changes state; this was agreed upon with the expert neurosurgeon as the minimum meaningful difference for treatment planning.

**Table 4:** Statistical relevance scores of the BN.

Factor	Statistical relevance score
Extracranial tumor	3.04
Primary Tumor Control	2.00
KPS	1.83
Neurological symptoms	1.12
Sex	0.94
Tumor Volume	0.74
Chemotherapy	0.32
Brain Metastases	0.10
Carcinomatous meningitis	0.06

Table 5 reports the comprehensive performance comparison including MAPE and R^2^ for all models and physicians. Note that ML metrics are means from repeated 5-fold CV and physician metrics are single evaluations on the same *N* = 350 test set. The BN achieved RMSE = 14.90 months and MAE = 8.96 months, which falls within the range of physician performance (RMSE: 12.95-14.36; MAE: 7.17-10.64), confirming that the BN provides prediction properties comparable to clinical experts. It is important to note that the ML baselines achieved lower RMSE and MAE than the BN (e.g., RF: RMSE = 10.84, MAE = 6.74 in repeated CV), indicating that data-driven models provide higher raw predictive accuracy. However, unlike these black-box ML models, the BN provides an explicit DAG that allows clinicians to visualize and trace the interrelationships between prognostic factors. Understanding why a model predicts a certain outcome is as critical as the prediction itself in the field of neurosurgery.

**Table 5:** Comprehensive performance comparison (RMSE, MAE, MAPE, R^2^).

Model/physician	RMSE	MAE	MAPE (%)	R^2^	Note
Proposed model					
BN	14.90	8.96	182.81	−0.231	Test set (*N* = 350)
ML baselines (repeated 5-fold CV)					
DT	11.00	6.88	263.97	0.234	Repeated 5-fold CV
RF	10.84	6.74	244.91	0.256	Repeated 5-fold CV
SVM	11.59	6.39	165.92	0.153	Repeated 5-fold CV
MLP	11.40	7.11	204.05	0.177	Repeated 5-fold CV
Physicians (single evaluation)					
Physician A	13.16	7.64	280.60	0.040	Single eval.
Physician B	14.36	10.64	504.33	−0.143	Single eval.
Physician C	13.28	7.17	206.67	0.023	Single eval.
Physician D	12.95	7.66	234.60	0.070	Single eval.
Physician E	13.62	7.66	161.09	−0.028	Single eval.
Physician F	13.28	8.80	363.12	0.023	Single eval.

ML model metrics are means from repeated 5-fold CV; Physician metrics are single evaluations on the same test set (*N* = 350).

## 4 Discussion

In this study, we presented an outcome prediction model for Gamma Knife radiosurgery using a Bayesian Network (BN) constructed from an expert physician's domain knowledge. While fully data-driven models such as deep neural networks offer high predictive performance, their clinical utility is often limited by a lack of explainability. Conversely, our approach, which directly encodes the clinical relationships from a physician's experience and knowledge into the model's structure, provides a naturally interpretable framework. To the best of our knowledge, this is the first study to model the OS of Gamma Knife radiosurgery using a BN explicitly built upon expert knowledge. From the perspective of cognitive neuroprosthetics, such knowledge-based systems can be interpreted as computational frameworks designed to augment human clinical reasoning.

The validity of this knowledge-based approach was elucidated from two critical perspectives. First, the model's interpretability was supported by a statistical relevance analysis, which confirmed that the inferred order of importance among predictive factors agreed with the expert physician's clinical intuition. This demonstrates that the BN provides not only a prediction but also an explainable rationale consistent with medical expertise. Second, our analysis of prediction error distributions revealed a notable distinction between physicians' predictions and the BN's. Using generalized hyperbolic distributions, we found that physicians' predictions exhibited significant skewness, indicative of cognitive bias (*p* = 0.031, Table 2). The BN's prediction error, conversely, was substantially less skewed. These results may imply that the BN formalizes expert knowledge while mitigating the individual cognitive biases that can influence clinical predictions. However, this mitigation was not sufficient to overcome deeper, shared limitations in prediction, particularly in cases where both human and model predictions systematically fail. In the context of cognitive augmentation, this suggests that the BN does not fully compensate for intrinsic limitations in human clinical reasoning. Goodness-of-fit of the GH distributions was verified by the KS test (Table 3). For Physicians A, D, E, and F, the KS test did not reject the fitted GH distributions under either the conventional threshold (*p* = 0.05) or the Bonferroni-adjusted threshold (α_adj_ = 0.0083). Physician B's parameters yield a near-degenerate distribution (σ = 1.67 × 10^−6^≈0, μ = 23.28 months); the KS test is not meaningful in this case. Physician C's parameters satisfy α≈β(22.856), causing a degenerate limit of the GH family.

It must be acknowledged that the BN did not outperform data-driven ML approaches in terms of RMSE and MAE. In the repeated cross-validation experiment, the best ML baseline (RF) achieved RMSE = 10.84 vs. the BN's RMSE = 14.90 on the physician test set–a difference of approximately 4 months that may be clinically meaningful given a median OS of 7 months. This interpretability-accuracy trade-off is well established in the clinical AI literature (Reddy, 2022; Amann et al., 2020). The primary contribution of this study is not to maximize predictive accuracy but to provide a transparent, knowledge-encoded framework suitable for cognitive augmentation, comparative error analysis, and iterative refinement of clinical knowledge structures. Nonetheless, this performance gap represents a recognized limitation that should be addressed in future studies through hybrid approaches combining the knowledge-based structure with data-driven parameter estimation.

Notably, a distinctive unilateral heavy-tailed shape was observed in the error distributions of both the physicians and the BN. This shared characteristic, indicating a systematic underestimation of outcomes for patients with exceptionally long survival, suggests that our model has captured a fundamental uncertainty inherent in the medical domain itself, rather than an artifact of the modeling process. Importantly, the fact that both human experts and the BN failed in the same subset of patients indicates that these errors arise from shared informational and representational constraints within clinical reasoning rather than from model-specific deficiencies. This points to the existence of uncharacterized factors or “unknown insights” at the frontier of current medical knowledge, which a purely data-driven model might obscure. From a neuroprosthetic viewpoint, this shared failure highlights a limitation: a cognitive prosthesis that reproduces, rather than compensates for, human clinical reasoning constraints may have limited augmentative value. In this sense, the BN does not merely serve as a predictive tool but as a lens to reveal the boundaries of current clinical knowledge.

Despite these promising results, this study has limitations. The number of factors included in the BN was relatively small. Because it uses discrete variables, the BN framework faces an intrinsic challenge with combinatorial explosion during both learning and inference as more variables are added. Thus, future study should focus on developing and incorporating learning algorithms designed to reduce computational load. Furthermore, to handle continuous data types, developing a mixed-model variant of the BN that integrates both discrete and continuous variables–an effort already underway in Bayesian learning frameworks with Gaussian random variables–would be a valuable extension. Furthermore, expanding the set of clinically relevant variables and incorporating newly identified factors may help address the unexplained variance observed in patients with unexpectedly long-term survival.

Based on these findings, a clear path forward is to develop this model into a clinical expert system to assist physicians in their practice. A key advantage of the BN framework is its inherent capacity for Bayesian updating. We envision a dynamic clinical decision-support system deployed on cloud servers that connects remotely located hospitals. In this system, clinical datasets would be regularly uploaded, and the outcome prediction model would be continually relearned as new data and evolving expert knowledge become available (Sutton et al., 2020; Musen et al., 2021; Bonczek et al., 2014; Alotaibi and Federico, 2017). With clinical data and physician knowledge, the plausibility and validity of the BN would continue to improve. Crucially, iterative updating of the underlying structure, guided by discrepancies between predicted and observed outcomes, may enable the discovery of previously unmodeled clinical relationships. Such a system would not be static but would improve over time, providing clinicians with an ever-more-refined and de-biased “second opinion”. Rather than simply improving accuracy, this framework emphasizes the progressive refinement of shared human-AI understanding. This iterative refinement process can be interpreted as an adaptive form of cognitive neuroprosthesis, in which discrepancies between human and model predictions guide the evolution of shared knowledge structures.

Ultimately, a hybrid approach that combines this knowledge-based framework with data-driven methods may offer the most robust solution, improving predictive accuracy without sacrificing the core explainability provided by the expert-derived structure. Close collaboration between data scientists and physicians, centered on such knowledge-based strategies, will be crucial to this endeavor. In conclusion, this study demonstrates that a BN constructed from expert knowledge serves as more than a prediction tool; it provides an interpretable model that reflects clinical reasoning estimated from statistical relevance analysis, quantifies systemic uncertainties, and reveals shared limitations between human and model-based prediction. Furthermore, it offers a principled framework for iteratively refining underlying knowledge structures by integrating additional expert knowledge and data, thereby supporting the development of next-generation cognitive neuroprosthetic systems for clinical decision-making.

## 5 Conclusion

This study demonstrated that a knowledge-based BN serves as more than a prediction tool for overall survival after Gamma Knife radiosurgery. Three principal contributions are reported: (1) to the best of our knowledge, this is the first study to develop a knowledge-based BN for OS prediction in Gamma Knife radiosurgery, achieving RMSE and MAE within the range of physician performance; (2) both the BN and six physicians exhibited a shared heavy-tailed error distribution, providing evidence that prediction failures arise from shared representational limits in current clinical knowledge rather than from model-specific deficiencies; and (3) the knowledge-based BN framework can be viewed as an adaptive cognitive neuroprosthetic system, in which discrepancies between human and model predictions can guide iterative refinement of clinical knowledge structures.

Future research directions include: (i) mixed discrete-continuous BN extensions to avoid information loss due to discretization; (ii) incorporation of additional clinical variables, such as molecular tumor markers; (iii) prospective multicenter validation; and (iv) Bayesian online updating within a real-time, cloud-based clinical decision-support system.

## Data Availability

The data analyzed in this study is subject to the following licenses/restrictions: The data supporting this study's findings are available from the corresponding author upon reasonable request. Requests to access these datasets should be directed to Yusuke Uchiyama, yusuke.uchiyama.dm@gmail.com.
